# *QuickStats*: Percentage* of Children and Adolescents Aged 5–17 Years Who Had Been the Victim of Violence or Witnessed Violence in Their Neighborhood,^†^ by Disability Status^§^ and Age Group — National Health Interview Survey, United States, 2022^¶^

**DOI:** 10.15585/mmwr.mm7301a6

**Published:** 2024-01-11

**Figure:**
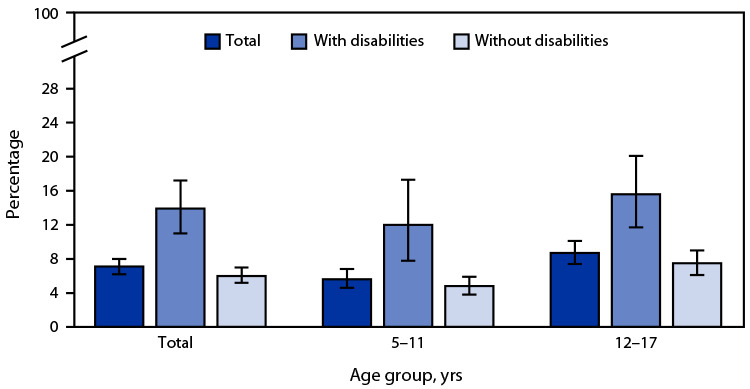
In 2022, 7.1% of children and adolescents aged 5–17 years had been the victim of violence or witnessed violence in their neighborhood. Percentages were higher among children and adolescents with disabilities (13.9%) than children and adolescents without disabilities (6.0%). This pattern was observed among children and adolescents aged 5–11 years (12.0% versus 4.8%) and those aged 12–17 years (15.6% versus 7.5%). Percentages increased with age among children and adolescents without disabilities from 4.8% among those aged 5–11 years to 7.5% among those aged 12–17 years. Percentages also increased with age for those with disabilities, but the observed difference (12.0% versus 15.6%) was not significant.

**Source:** National Center for Health Statistics, National Health Interview Survey, 2022. https://www.cdc.gov/nchs/nhis.htm

